# Physical activities of Patients with adolescent idiopathic scoliosis (AIS): preliminary longitudinal case–control study historical evaluation of possible risk factors

**DOI:** 10.1186/s13013-015-0029-8

**Published:** 2015-02-18

**Authors:** Marianne E McMaster, Amanda Jane Lee, R Geoffrey Burwell

**Affiliations:** Scottish National Paediatric, Spine Deformity Centre, Royal Hospital for Sick Children, Edinburgh, EH9 1LF UK; Centre for Spinal Studies and Surgery, Queen’s Medical Centre Campus, Nottingham, UK; Medical Statistics Unit, University of Edinburgh, Teviot Place, Edinburgh, EH8 9AG UK

**Keywords:** Scoliosis, Physical activities, Risk factors, Etiology, Swimming pools, Toe touching

## Abstract

To our knowledge there are no publications that have evaluated physical activities in relation to the etiopathogenesis of adolescent idiopathic scoliosis (AIS) other than sports scolioses. In a preliminary longitudinal case–control study, mother and child were questioned and the children examined by one observer. The aim of the study was to examine possible risk factors for AIS. Two study groups were assessed for physical activities: 79 children diagnosed as having progressive AIS at one spinal deformity centre (66 girls, 13 boys) and a Control Group of 77 school children (66 girls, 11 boys), the selection involving six criteria.

A structured history of physical activities was obtained, every child allocated to a socioeconomic group and examined for toe touching. Unlike the Patients, the Controls were not X-rayed and were examined for surface vertical spinous process asymmetry (VSPA). Statistical analyses showed progressive AIS to be positively associated with social deprivation, early introduction to indoor heated swimming pools and ability to toe touch. AIS is negatively associated with participation in dance, skating, gymnastics or karate and football or hockey classes, which might suggest preventive possibilities. There is a significantly increased independent odds of AIS in children who went to an indoor heated swimming pool within the first year of life (odds ratio 3.88, 95% CI 1.77-8.48; p = 0·001). Furthermore fourteen (61%) Controls with VSPA compared with 9 (17%) Controls without VSPA had been introduced to the swimming pool within their first year of life (P < 0.001). Early exposure to indoor heated swimming pools for both AIS and VSPA, suggests that the AIS findings do not result from sample selection.

## Introduction

This paper reports a preliminary longitudinal study of the physical activities of children obtained historically for Patients with progressive adolescent idiopathic scoliosis (AIS) and Controls. For reasons given in Appendix I, this text describing the research is a full account of that previously presented [1-3]*.*

The cause of AIS is unknown, it is generally considered to be multifactorial in origin and thought to have separate factors for curve initiation and progression [4-6]*.* There is support for the view that genetics stipulates the course of AIS [7-9]. Wynne-Davies [10]*,* examining the etiology of some common skeletal deformities including infantile idiopathic scoliosis, concluded that all are likely to have a common multifactorial genetic background associated with differing intrauterine or postnatal environmental factors. Monozygotic twins have been used to demonstrate the role of environmental factors in determining complex diseases and phenotypes, but the true nature of the phenotypic discordance remains poorly understood [11,12]. In the last decade, sporadic reports have suggested environmental factors are involved in the etiopathogenesis and phenotypic expression of AIS [12].

To our knowledge there are no publications that have evaluated physical activities in relation to the etiopathogenesis of AIS other than sports scolioses [13,14] and trunk asymmetries with swimming [15]. The aim of the study was to examine possible risk factors for AIS; and in particular to test whether one or more of a variety of physical activities is related to the presence or absence of progressive AIS.

## Subjects and methods

### Selection of subjects

One observer (MM) obtained the histories and undertook the physical examinations. Patients and Controls were excluded if they did not fulfil the Six Criteria for selection and/or their family history was unknown, namely: being at boarding school or university and were unable to attend with their mother; from a one parent family; was adopted or a parent had been adopted (Appendix II).

### Six criteria for further selection of subjects

Patients with AIS and Controls selected for the study were defined by Six Criteria:-

1. Born full term.
2. Fed well - no feeding problems during 1st year.
3. Achievement of normal milestones i.e. walking and talking.
4. No hospital referrals – excluding sports injuries.
5. No back pain (before diagnosis in Patient Group).
6. No family history of scoliosis.

#### Patients

The Patients were diagnosed as having AIS by one spine surgeon. Initially, 100 consecutive Patients were referred but, due to requirements for both the structured questioning and selection by Six Criteria 79 Patients (girls 66, boys 13) were included in the study (mean age 15.1 years).

### Controls

One hundred consecutive Controls were volunteers from three schools in north and south Scotland, and 12 Controls who had recently joined a tennis centre. After applying the Six Selection Criteria, 77 Controls (girls 66, boys 11) were included in the study (mean age 14.7 years).

#### Physical examinations

The Patients and Controls were examined before questioning.

The Patients were referred for the primary purpose of an Integrated Shape Imaging System (ISIS) scan (which is an optical topographic system for measuring back shape in 3-dimensions), although the ISIS data is not presented here.

ISIS scanning requires markers to be placed on the spinous processes defined along the length of the spine from the vertebra prominens to L5 and it was the basis of this technique which was adapted to assess surface spinal asymmetry of the Controls.

### Patients – severity

**Figure 1:**
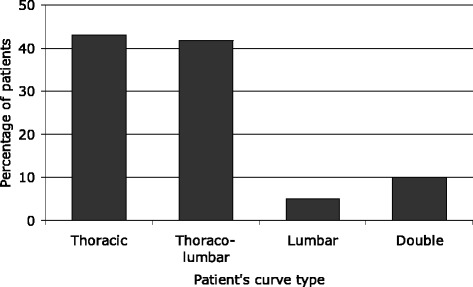
**Frequency distribution of the curve types of the AIS patients.**

Figure 1 shows the curve types of the Patients. The mean Cobb angle was 45 degrees (range 10–78). All curves were progressive and 62 patients subsequently had a spinal arthrodesis.

### Controls *-* surface vertical spinous process asymmetry (VSPA)

Standing in an erect position to assess spinal symmetry/asymmetry of the Controls, the observer first identified the vertebra prominens [16]*.* The observer held her left thumb on the vertebra prominens throughout the examination. Then, using her right thumb to palpate each spinous process until reaching L5 and without using skin marks, markers or a plumb line, she looks for both lateral spinal curvature and/or axial spinal rotation to judge the presence or absence of surface spinal asymmetry relative to a visual vertebra prominens - L5 line, termed here *vertical spinous process asymmetry* (VSPA). The examination was repeated several times to ensure accuracy.

Finally, both thumbs were placed on the posterior superior iliac spines, Dimples of Venus, to assess leg length inequality and a level pelvis.

#### Reproducibility

In 54 hospital staff volunteers (females 52, males 2), none known to have scoliosis, in examinations repeated after intervals, the Observer (MM) detected VSPA in 2 but not consistent in one of the females. The palpation of spinous processes was more difficult than in the children because of subcutaneous fat.

The forward bending test was not used for the Controls (Appendix III) and they were not examined neurologically.

### Toe touching

Every subject (Patients and Controls) was asked to touch their toes with straight knees.

### Structured questioning

After the examination, a structured interview was obtained in both groups. One investigator (MM) undertook the interviews, which followed the same rigid structure for both groups. A history of each child’s physical activities was obtained from the mother and child, who were questioned together for a mean time of 47 minutes. The same rigid structure of questioning was adhered to in both the Patient and Control Groups

The questioning was divided into pre-school regular outdoor activities and after-school and weekend activities that were undertaken at least once a week for a minimum of three terms (1 year). The mother was asked to recall all physical activities that her child had participated in before 5 years of age, starting before her child could walk. Use of toys, tricycles, and bicycles with stabilizers were all recorded, with resounding similarity between both groups.

After 5 years of age, only after-school and weekend activities were recorded that were undertaken at least once a week for a minimum of three terms (1 year) under the following headings: dancing, gymnastics, karate, swimming, football, hockey, and rowing. Horse riding was included but was rarely a weekly activity.

Although 12 Controls were from a tennis centre, none had participated in tennis for the minimum of three terms. Cycling was a regular activity undertaken in neighbourhood by both Patients and Controls before 10 years of age. Skating (roller blade and/or ice) was also a neighbourhood activity.

Winter and summer holiday activities *i.e.* skiing were recorded but not analyzed, since these activities were only undertaken for a maximum of 6 weeks.

### Socioeconomic status

The socioeconomic status of Patients and Controls was established by Carstairs’ deprivation score [17] using the Subjects’ postcode. This method enabled the children to be given a score of 1–5 (most affluent to most deprived).

### Statistical analyses

The χ^2^ test (or Fisher’s exact test) was used to compare two percentages, and odds ratios (OR) were calculated with 95% confidence intervals (CI).

Stepwise logistic regression was used to examine which factors were significant independent predictors of AIS. The variables considered for entry into the model were sex, Carstairs’ deprivation score (most affluent versus the remaining four groups), whether the child could swim by the age of 10 years, whether the child first attended the swimming pool (on at least eight occasions) before their first birthday, and whether the child participated in the following activities: gymnastics or karate, skating, horse riding, football, or hockey.

Attendance at gymnastics/karate and football/hockey were grouped together, because participation differed between the sexes. For this reason attendance at dance classes was not included in the logistic regression. Two separate models were produced, one which considered the temporary activities of skating and horse riding, and the other which excluded these activities from consideration. Another separate model examined the effects of both toe touching and introduction to the swimming pool within the first year of life, as toe touching had not been included in the main models as it could have been secondary to AIS.

## Ethics

This study was undertaken before ethics approval was required for such research and was prosecuted in accordance with the Helsinki Declaration.

## Findings

Table 1 shows the relationship between each possible risk factor and group (Patient or Control).

**Table 1:** **Association between risk factor and asymmetry**

**Risk factor**	Percentage with risk factor*		**χ** ^**2**^ **test statistic or Fisher’s exact test (p-value)**	**OR (95% CI)**
	**Cases**	**Controls**		
Carstairs’ deprivation score			17.7 (0.001)	
1	38	62		1.00
2	28	6		7.04 (2.41 – 20.6)
3	18	9		3.20 (1.16 – 8.83)
4 or 5	16	22		1.22 (0.52 – 2.87)
Carstairs’ deprivation score			0.16 (0.689)	
1-2	66	69		1.00
3-5	34	31		1.15 (0.59 – 2.24)
Carstairs’ deprivation score			9.56 (0.002)	
1	38	62		1.00
2-5	62	38		2.70 (1.42 – 5.16)
Asymmetry in mother’s back	1	4	----- (0.364)^+^	0.32 (0.03 – 3.11)
Hamstring muscles not tight/ability to touch toes	80	60	6.49 (0.011)	2.65 (1.30 – 5.41)
Poor social skills	1	3	0.00 (0.982)	0.48 (0.04 – 5.41)
No previous cycling	0	0	---	---
No previous attendance at dance classes (girls only)	44	26	4.04 (0.045)	2.26 (1.08 – 4.71)
No previous gymnastics/karate	76	53	7.84 (0.005)	2.77 (1.40 – 5.49)
No previous skating	73	32	24.6 (<0.001)	5.74 (2.88 – 11.5)
No previous horse riding	82	60	8.58 (0.003)	3.13 (1.50 – 6.53)
No previous playing football/hockey	65	55	1.23 (0.267)	1.52 (0.80 – 2.89)
Introduced to swimming pool within first year of life	58	31	10.5 (0.001)	3.08 (1.60 – 5.94)
Ability to swim by age 10 years	56	56	0.00 (0.999)	0.99 (0.53 – 1.87)
Introduced to public pool within first year (≥8)	57	30	10.6 (0.001)	3.11 (1.61 – 6.02)
Introduced to public pool within first year (≥12)	54	29	9.69 (0.002)	2.99 (1.54 – 5.80)
Introduced to public pool within first year (≥24)	34	23	1.72 (0.190)	1.70 (0.84 – 3.44)

*All patients and controls completed every question, therefore the results are presented for 79 cases and 77 controls (with the exception of dancing classes which only applied to girls – 66 patients and 66 controls).

^+^Fisher’s exact test used rather than χ^2^ test due to small numbers.

### Socioeconomic status

Subjects with a score of 2, 3 or 2–5 combined on the Carstairs’ deprivation score all had significantly increased odds of having AIS compared with those with a Carstairs’ deprivation score of 1 (most affluent), although there was no difference for those with a Carstairs’ deprivation score of 4–5 combined. Although when the two most affluent groups were combined, there was no significant increase in the odds of AIS.

### Indoor heated swimming pools

There is a significantly independent increased odds of AIS (OR 3.08, 95% CI 1.60 to 5.94) in children who went to an indoor heated swimming pool within the first year of life with 58% of Patients having been introduced to an indoor heated swimming pool within their first year compared with 31% of Controls.

### Physical activities

Children who did not attend dance (girls only) or gymnastics or karate classes, or regularly participated in horse riding and skating had higher odds of having AIS than did those who participated in these activities. There was no statistically significant association for previous regular football or hockey.

### Toe touching

A significantly higher proportion of Patients than Controls could touch their toes (80% *vs* 60%). Children who could touch their toes had more than 2·5 times the odds of having AIS compared with those who could not.

### Swimming

There was a statistically significant difference in the age at which Patients and Controls regularly attended a heated swimming pool (Fisher’s exact test, p = 0.0010) with Patients introduced earlier than Controls (Table 2). Furthermore, Controls who had VSPA had also regularly attended an indoor heated swimming pool earlier than those Controls without VSPA (Fisher’s exact test, p < 0.0001). Although there was no statistically significant difference in the percentages of Patients and Controls who could swim by age 10 years (χ^2^ test, p = 0.99), there was a difference among the Controls with and without VSPA (χ^2^ test, p = 0.0135).

**Table 2:** **Age at which children were first introduced to an indoor heated swimming pool**

**Age regular swimming/attending pool**	**AIS patients**	**Controls**	**Controls with VSPA**	**Controls without VSPA**
	**Number (%)**	**Number (%)**	**Number (%)**	**Number (%)**
1st year	46 (58.2)	24 (31.2)	16 (64.0)	8 (15.4)
2nd year	6 (7.6)	9 (11.7)	1 (4.0)	8 (15.4)
3rd year	4 (5.1)	3 (3.9)	2 (8.0)	1 (1.9)
4th year	4 (5.1)	5 (6.5)	2 (8.0)	3 (5.8)
5th year	7 (8.9)	7 (9.1)	1 (4.0)	6 (11.5)
6th year	2 (2.5)	14 (18.2)	3 (12.0)	11 (21.2)
7th year or later	7 (8.9)	15 (19.5)	0 (0.0)	15 (28.8)
Never in pool	3 (3.8)	0 (0.0)	0 (0.0)	0 (0.0)
Can swim by age 10 years	44 (55.7)	43 (55.8)	19 (76.0)	24 (46.2)

Three of the Patients had never been to an indoor heated swimming pool but each of the Controls had.

### Independent factors associated with AIS

Using stepwise logistic regression, the following factors were significant independent predictors of AIS:

1. Carstairs’ deprivation score (1 most affluent versus 2–5);
2. introduction to a pool within the first year of life (≥eight occasions);
3. no previous skating; and
4. no attendance at gymnastics or karate classes (Table 3).

**Table 3:** **Significant independent predictors of asymmetry (temporary activities included but touching toes excluded) using Carstairs 1 versus 2–5 and eight swimming visits**

**Significant independent predictor**	**Parameter estimate (standard error)**	**Wald statistic (p)**	**OR (95% CI)**
Constant	−7.47 (1.35)	30.7 (<0.001)	---
No previous skating	1.63 (0.39)	17.6 (<0.001)	5.09 (2.38–10.9)
Introduced to swimming pool within first year (≥8)	1.36 (0.40)	11.5 (0.001)	3.88 (1.77–8.48)
Carstairs’ Deprivation score (2–5 = most deprived)	1.02 (0.39)	6.96 (0.008)	2.78 (1.30–5.96)
No previous gymnastics/karate	1.03 (0.41)	6.33 (0.012)	2.79 (1.25–6.20)

Children who were introduced to a pool within the first year of life had 3·9 times the odds of having AIS than did those who were not introduced to the pool. Whereas children who had a Carstairs’ deprivation score of 2–5, had 2.8 times the odds of having AIS compared with those in the most affluent category (score of 1). Children who had no previous skating and no previous gymnastics or karate were 5.1 and 2.8 times the odds respectively of having AIS compared with those who participated in these activities.

A further model (Table 4) was derived with stepwise logistic regression that excluded skating and horse riding, since these activities were regarded as temporary. The model was similar to that which included all activities except that no previous skating was replaced with no previous football or hockey. The odds of having AIS among those with a Carstairs’ deprivation score 2–5 and no previous gymnastics or karate increased slightly (to 3.3 and 3.5 respectively), and there was 2.1 times the odds of AIS among those who did not undertake football or hockey compared with those who did participate in these activities.

**Table 4:** **Significant independent predictors of asymmetry (temporary activities and touching toes excluded) using Carstairs 1 versus 2–5 and eight swimming visits**

**Significant independent predictor**	**Parameter estimate (Standard error)**	**Wald statistic (p)**	**OR (95% CI)**
Constant	−6.73 (1.32)	25.9 (<0.001)	---
Introduced to swimming pool within first year (≥8)	1.37 (0.38)	13.1 (<0.001)	3.91 (1.87–8.20)
No previous gymnastics/karate	1.26 (0.39)	10.7 (0.001)	3.53 (1.66–7.53)
Carstairs’ Deprivation score (2–5 = most deprived)	1.18 (0.37)	10.4 (0.001)	3.27 (1.59–6.72)
No previous football/hockey	0.76 (0.38)	4.05 (0.044)	2.14 (1.02–4.49)

### Early introduction to swimming pool and toe touching

Of the 45 Patients who had been taken to a public swimming pool in their first year of life, 39 (87%) could touch their toes at time of examination.

Whereas 23 Controls who were taken to a public swimming pool before 1 year of age, 14 (61%) could touch their toes.

Using both early introduction to swimming and toe touching together in a logistic regression model, both were significant independent predictors of AIS. There was 2.86 (95% CI 1.46 to 5.60) times the odds of AIS for those introduced to the pool (≥8 occasions) within their first year compared with those who were not introduced to the pool (p = 0.002); and there was 2.35 (95% CI 1.13 to 4.91) times the odds of AIS for those who could touch their toes compared with those who could not (p = 0.023).

## Discussion

Swimming has long been considered as beneficial for children diagnosed with scoliosis.

### AIS and control samples

The samples are not large but were considered adequate for the statistical tests applied in a preliminary study but with the size of our samples, we cannot exclude the possibility of random variation contributing to the findings (Appendix II). The mean ages of the samples are not significantly different; the greater variance of the AIS group did not affect the collection of historical evidence. The selection of 12 Controls from a tennis centre might have affected the findings, since they could have been more active, though none of these children had played tennis every week for a period of three terms.

### Structured questioning

The structured history taking of physical activities helped with the problem of obtaining reliable memories over several years. The history was always procured from the mother often accompanied by the father. The observer (MM) was impressed by the capacity of the mothers to recall the early years of their child’s physical activities; less so for later years where the child was noted to have a clear recollection.

### Independent factors associated with AIS

Five significant independent predictors for AIS were:

1. increased deprivation;
2. introduction to a swimming pool within the first year of life (≥eight occasions)
3. no previous skating (model including temporary activities);
4. no attendance at gymnastics, or karate classes; and
5. no attendance at football or hockey classes (model excluding temporary activities).

These are predictive in the statistical models and only possible risk factors for AIS while awaiting the results of future research.

### Association of AIS in more deprived socioeconomic groups

Significantly increased odds of AIS was found in more deprived socioeconomic groups (score 2–5) than in the most affluent group, score 1. The findings are inconsistent with those reported by Ryan et al. [18] who reported that AIS was more common in high than in low socioeconomic groups. Carstairs’ deprivation score is not a precise indicator of socioeconomic group as it is defined at a geographical area level (postcode) rather than at an individual level. This could be one reason for the inconsistent findings.

However, since an association between Carstairs’ deprivation score and participation in various activities was noted, it was necessary to include Carstairs’ deprivation into the model (as it was a confounder) so that assessment of which factors are significant independent predictors of AIS can be considered, i.e. factors that have an association with AIS after taking deprivation into account.

### Association of AIS with introduction to a swimming pool within the first year of life

The association of AIS with early exposure to indoor heated swimming pool, if not due to sampling and chance, does not prove a causative link between AIS and such exposure, but only the unproven possibility of such a link for which an hypothesis has been formulated [19]*.*

### Two asymmetries of the spinal column

Two asymmetries of the spinal column were identified namely, in (a) the Patients with AIS with spinal radiographs and (b) the Controls with VSPA.

The finding of VSPA associated with early exposure to indoor heated swimming pools was unexpected and an original finding. This VSPA method is not generally used by spine surgeons in such detail to assess lateral spinal curvatures of the spine. The observer (MM) used this method as a result of 17 years’ experience with ISIS scanning where the physical identification of spinous processes is needed in the preparation for ISIS scanning.

### What does the presence of VSPA indicate?

VSPA was found to be common in the Controls (30%). As the Controls were not referred for X-ray examination due to ethical restriction, their Cobb angles of the VSPAs are not known. Besides mild structural lateral spinal curvatures VSPAs may express pelvic tilt scoliosis and postural scoliosis. The Controls were not examined neurologically. In the year after they were examined, seven of the Controls with VSPA were referred to the scoliosis clinic by their general practitioners.

### Not the result of sample selection?

The association of attendance as infants to indoor heated swimming pools with each of AIS and VSPA, suggests that this association for AIS children did not result from sample selection.

### Separate initiating and progressive factors for AIS?

In the Control group, strong evidence of an association between VSPA and early indoor pool attendances, is the finding that 61% of those with VSPA had been taken to an indoor heated swimming pool as infants. The evidence is consistent with the speculation that VSPA relative to AIS may express either:

- an initiating factor(s) in swimming pool ambience for AIS with other factors needed for curve progression; or
- a weaker initiation.

### Environmental factor(s) for AIS and VSPA?

The indoor heated swimming pool association with the later expression of AIS and VSPA raises the speculation that there might be other environmental factors acting in the first year of life to initiate the later expression of AIS that differ around the world, with an environmental effect suggested from research in Scotland (see below ethnicity, latitude and swimming pools).

### Number of swimming pool visits and AIS

There were increased odds of AIS for children who were introduced to the swimming pool within the first year of life if attendance was defined as eight or more or 12 or more visits, but not if attendance was defined as 24 or more visits. It is not clear why this might be the case, and this needs to be examined in more detail in further research.

### Association of reduced exposure to some physical activities

In the AIS group, the reduced exposure to eg. gymnastics, karate, skating, and dance classes of girls suggests either:

- the sampling of more active Controls, and/or
- the relatively reduced exposure to these physical activities predisposed the children to progressive AIS, and/or
- children with AIS feel less confident with physical activities and do not participate as much as other children.

Point (b) raises the possibility that increased physical activities as possible therapy, may protect against AIS by involving neuromuscular feedback mechanisms common to all joints [20]*.*

Herman and co-workers [21] suggested that idiopathic scoliosis involves visuo-spatial perception impairment. If so, perhaps the introduction of specific physical activities to more members of a community by facilitating the onset of balance skills within the framework of posture might prevent some children from getting progressive AIS.

### Association of toe touching with AIS

After correction for deprivation score, a significant association between AIS and toe touching (positive risk factor) was recorded compared with early swimming (positive risk factor), and not doing various sporting activities, which potentially has therapeutic implications.

Toe touching involves separate contributions from the hip, lumbar, and thoracic spines in the sagittal plane [22]. Joint laxity is associated with AIS [23] and although the hypothesis that a defect of connective tissues is a causative factor, it is not established.

The increased ability to toe touch in Patients with AIS could be associated with the physique of the children or with factors such as joint laxity that are associated with the cause of the spinal deformity [22]. Taylor and Melrose [24] support the hypothesis that collagen abnormalities in the intervertebral disc have a contributory role to the development and evolution of the curvature.

### Ethnicity, latitude and swimming pools

Smyrnis and colleagues [25] noted an increased prevalence of spinal asymmetry in children with fair hair and blue eyes in their study of about 3500 Greek schoolchildren.

Grivas and colleagues [26] have shown the prevalence of AIS according to geographical location in the northern hemisphere, with the incidence decreasing as the latitude approaches the equator. The investigators suggest that these data might represent real environmental, geographical, genetic, or racial factors for this disease. Environmentally, it seems reasonable to link the geographical latitude of AIS to the location of indoor heated swimming pools - and Scotland has a northern climate. The heating of a pool and its enclosure is determined by the prevailing outdoor temperature.

### Children, swimming pools, disease, conditions and pollution

How swimming in an indoor heated swimming pool early in the first year of life may be associated later in life with the development of AIS and VSPA is unknown. It may be due to sampling differences, or be related to causation. Sampling seems less likely by the finding that the early swimming pool exposure relates to both AIS and the VSPA of healthy children. More research is needed.

Other research has shown detrimental exposure to chemicals that are found in swimming pools**.** No data on chemicals in the pool ambience during the period of this study are known to us.

Bessac and colleagues [27] found that that chlorine triggers nerve endings not only in the lungs but also in peripheral tissues of mice. Nystad and co-workers [28] have noted that taking a baby swimming increases infections of the respiratory tract and middle ear. In a study assessing children exposed to the air of indoor-chlorinated pools, Bernard and colleagues [29] reported a link to asthma and chronic airway inflammation, which they suggest could be due to an irritant gas trichloramine (nitrogen trichloride) contaminating the air of indoor-chlorinated pools. Chlorine reacts with bodily proteins to form chloramines, the most volatile and prevalent of which in the air above swimming pools is nitrogen trichloride [30]. Waterborne pathogens are destroyed by chlorine; however, when sweat, urine, and faecal bacterial levels overpower the chlorine hypochloride, a breakdown product such as trihalomethane is formed [31]. No child with asthma and receiving prescribed medication for their disease would have been included in our studies as either a Patient or a Control.

Grandjean and Landrigan [32] reported that one in six children has some type of developmental disability, usually involving the nervous system, and that developing brains are much more susceptible to toxic chemicals than those of adults. Furthermore, they believed that identifying the effects of industrial chemical pollution is difficult because patients might not produce symptoms for several years or even decades. The article concluded “The combined evidence suggests that neurodevelopmental disorders caused by industrial chemicals has created a silent pandemic in modern society.”

### Barker hypothesis

Barker and colleagues [33] showed that the origins of important chronic diseases in adult life might lie not only in the intrauterine environment but also in early postnatal life. Whether or not this concept is applicable to the findings reported here is unknown.

## Conclusions

1. Statistically significant associations were found between AIS and each of social deprivation, early introduction to an indoor heated swimming pools, not participating in some sporting activities and ability to toe touch. These are possibly positive and negative risk factors for AIS, which may have therapeutic potential.
2. The association of AIS with early exposure to indoor heated swimming pool, if not due to sampling and chance, does not prove a causative link between AIS and such exposure, but only the unproven possibility of such a link for which an hypothesis has been formulated
3. Early exposure to indoor heated swimming pools was also associated with vertical spinous process asymmetry (VSPA).
4. VSPAs may express mild structural lateral curvatures, pelvic tilt scoliosis and postural scoliosis.
5. The association of attendance as infants to indoor heated swimming pools with each of AIS and VSPA, suggest that this association for AIS children may not result from sample selection.
6. The evidence is consistent with the speculation that VSPA relative to AIS may be an expression of either: (a) an initiating factor(s) in swimming pool ambience for AIS with other factors needed for curve progression; or (b) a weaker initiation.
7. The speculation suggested that there might be other environmental factors acting in the first year of life to initiate the later expression of AIS, that differ around the world
8. Confirmation of these preliminary findings is needed.

## Appendix I

This paper provides a full account of that published privately in abridged form with one Table by the International Research Society of Spinal Deformities meeting at the University of British Columbia, Canada in 2004 [1]*.* The research led to a further publication by the writer [4].

## Appendix II

### Patients

The Patient Group consisted of 79 adolescents. Of which 66 (84%) were girls (mean age 15·1 years, SD 3 · 0, range 10 years 9 months–25 years 3 months) and 13 (16%) were boys (mean age 14 years 10 months, SD 1 · 8, range 11 years 2 months–18 years 7 months at time of interview.

### ISIS scanning

ISIS (Integrated Shape Imaging System) is an optical topographic system for measuring back shape in 3-dimensions. The lateral spinal curvature measured by ISIS equates with the radiological Cobb angle. ISIS is very unlikely to reliably detect scolioses below 10 degrees (Shannon T, personal communication). Cobb angles below 10 degrees is viewed as normal.

### Controls

77 consecutive Controls: all of whom met the six inclusion criteria. 66 (86%) were girls (mean age 14 years 6 months, SD 1·1, range 11 years 9 months–16 years 11 months) and 11 (14%) boys (mean age 15 years 2 months, SD 1·9, range 12 years 6 months–18 years at the time of interview).

### Age of patients and controls

There was a significant difference in the variance in the ages among the Patients (SD 2.86 years) compared with Controls (SD 1.22 years) as Patients ranged from 10.8 to 25.3 years and Controls ranged from 11.8 to 18.0 years. Using a t-test with unequal variance, there was no statistically significant difference in the mean age 1.13, df = 106, p = 0.26) of Patients (mean age 15.1 years) and Controls (mean age 14.7 years).

### Sample sizes

The study is sufficiently powered statistically to examine the primary aim of examining early exposure to swimming in relation to AIS. With an anticipated exposure to early swimming among the Controls of 30% and with an increased risk of 3 or more, testing at the 5% significance level with 80% power, one would require 55 Patients and 55 Controls. With 90% power, one would require 73 Patients and 73 Controls. The study had 79 Patients and 77 Controls so we had more than 90% power which is the maximum generally used in sample size calculations.

### Ethnicity

The children in both the Patient and Control Groups were Caucasian, apart from one female patient. This patient was non-white judged by the third generation, but because of the weakness of this factor was not excluded from the study.

### Obesity

Six children were considered overweight as defined by a fat fold below the rib cage (two Patients, four Controls). Their physique was not deemed to inhibit toe touching or the observer’s ability to palpate the spinous processes.

## Appendix III

### Forward bending test (FBT)

The FBT, quantified with the Scoliometer [34] has never been used in the Edinburgh Spinal Deformity Centre. This is because: [1] the FBT provides a visual and/or digital estimate of axial trunk rotation and only indirectly of lateral spinal curvature, and [2] long experience in applying markers to each spinous processs from vertebra prominens to L5 preparatory to ISIS scanning, led to the observer (MM) to apply this technique to the Controls.
