# Erratum to: Physical activities of Patients with adolescent idiopathic scoliosis (AIS): preliminary longitudinal case–control study historical evaluation of possible risk factors

**DOI:** 10.1186/s13013-016-0070-2

**Published:** 2016-03-18

**Authors:** Marianne E. McMaster, Amanda Jane Lee, R. Geoffrey Burwell

**Affiliations:** Scottish National Paediatric Spine Deformity Centre, Royal Hospital for Sick Children, Edinburgh, EH9 1LF, UK; Medical Statistics Unit, University of Edinburgh, Teviot Place, Edinburgh, EH8 9AG, UK; Centre for Spinal Studies and Surgery, Queen’s Medical Centre Campus, Nottingham, UK

The ethics statement in this article [1] incorrectly states that the study was undertaken before ethics approval was required for such research. Contrary to this statement, South East Scotland Research Ethics Service has confirmed that an NHS ethics committee was in existence at the time of the study during the 1990s and that ethics approval would have been required for the NHS aspects of this study. South East Scotland Research Ethics Service has advised that the study would most likely have been given a favourable opinion if submitted for ethics approval but are unable to give retrospective approval. Given the time when the study was conducted, the non-invasive nature of the study, the opinion of the South East Scotland Research Ethics Committee and the potential public health implications of the findings, the Editor and Publisher have decided to take no further action.

In addition, there is an error in the reference list of this article [1]. The article cited as reference 19 should have been included in the reference list between references 3 and 4.

The first paragraph of the introduction section should include a citation to the article listed as reference 19 to read:

*This paper reports a preliminary longitudinal study of the physical activities of children obtained historically for Patients with progressive adolescent idiopathic scoliosis (AIS) and Controls. For reasons given in Appendix I, this text describing the research is a full account of that previously presented [1–3, 19]*.

Appendix I should read:

*This paper provides a full account of that published privately in abridged form with one Table by the International Research Society of Spinal Deformities meeting at the University of British Columbia, Canada in 2004 [1]. The research led to a further publication by the writer [19].*
